# Editorial: Neuro-cognitive Architecture of Numerical Cognition and Its Development

**DOI:** 10.3389/fnhum.2021.670460

**Published:** 2021-04-28

**Authors:** Elise Klein, Reuven Babai, Anja Ischebeck, Korbinian Moeller

**Affiliations:** ^1^Université de Paris, LaPsyDÉ, CNRS, Sorbonne Paris Cité, Paris, France; ^2^Leibniz-Institut fuer Wissensmedien, Tuebingen, Germany; ^3^Department of Mathematics, Science and Technology Education, Tel Aviv University, Tel Aviv, Israel; ^4^Institute of Psychology, University of Graz, Graz, Austria; ^5^Mathematics Education Centre, School of Science, Loughborough University, Loughborough, United Kingdom

**Keywords:** numerical cognition, numerical development, number processing, neural correlates, neuro-cognitive plasticity, developmental dyscalculia, numerical training

Living in our modern societies requires being numerically literate not only in everyday life (e.g., reading the clock, dealing with money, etc.) but also in educational (e.g., mathematics and science classes) and professional contexts (e.g., accounting but also crafts). Hence, numerical and mathematical skills were repeatedly observed to predict not only occupational success (e.g., Ritchie and Bates, 2013) but also more general life prospects (Parsons and Bynner, 2005). However, insufficient numerical and mathematical skills still affect a considerable share of students—even in developed Western societies as reflected in international comparison studies such as PISA (OECD, 2016).

In recent years, numerical cognition research made considerable progress in describing and understanding cognitive processes and succeeded in specifying the neural architecture underlying numerical cognition. On the basis of these developments, we invited empirical and theoretical contributions for a Research Topic on the *Neuro-cognitive Architecture of Numerical Cognition and Its Development*. We are grateful to all authors for their high-quality contributions, the reviewers for their constructive feedback and helpful suggestions during the interactive peer-review, and the publisher's editorial team for their excellent support.

The 15 contributions to our Research Topic from internationally leading groups cover different aspects of numerical cognition in children, adolescents and adults, as well as its theoretical background. The applied methods run the gamut from behavioral to neuroimaging studies, from cross-sectional to longitudinal and intervention studies. The different contributions nicely illustrate that *numerical cognition* is not a unitary and closely circumscribed construct, comprehensively accounting for the evidence observed. Instead, the empirical and theoretical contributions indicate, that there are domain-specific aspects of numerical cognition (e.g., number sense, arithmetic, spatial-numerical associations, etc.) that are influenced by domain-general abilities or traits (e.g., visuospatial abilities, self-regulation, and math anxiety).

A first set of studies investigated domain-specific and domain-general predictors of later numerical or mathematical skills. In a longitudinal behavioral study, Finke et al. observed that early non-symbolic numerical skills predicted later arithmetic skills by facilitating the acquisition of symbolic number processing. The authors concluded that non-symbolic numerical skills are foundational skills to mathematical development. Braeuning et al. proposed a multifactorial structure of early numeracy, which they tested using a confirmatory factor analysis approach on longitudinal large-scale assessment data. The authors identified four specific basic numerical skills (i.e., number sense, arithmetic, patterning/geometry, and data analysis/statistics) that remained stable from 5 to 6 years of age and thus seem to serve as building blocks for further numerical development. Schild et al. evaluated the causal influence of finger-based numerical training on initial arithmetic skills in 5 year-old children. They did not observe an advantage for the finger-based training, which they attributed to domain-general processes such as sequencing that were also required in the control training. In a cross-cultural behavioral study, Nemati et al. found an association between domain-general personality traits such as self-regulation and mathematical performance that was similar for German and Iranian students. Finally, in a commentary on the idea of a mental number line in human newborns as proposed by Di Giorgio et al. (2019), Felisatti et al. postulated the Brain's Asymmetric Frequency Tuning (BAFT) hypothesis. The BAFT hypothesis emphasizes the relevance of spatial frequencies (SFs) for spatial numerical associations and embodied numerical representations in humans. The authors posited that spatial-numerical associations in newborns (1) may be driven by absolute and relative spatial frequency processing, (2) generalize across cultures and species, and (3) may be different in newborns predisposed to atypical numerical development.

The relationship between brain structure and numerical processing abilities was investigated by two contributions: McCaskey et al. used voxel-based morphometry in a longitudinal study comparing children with developmental dyscalculia with typically developing children. The authors observed that dyscalculic children between 8 and 11 years of age had persistently reduced gray and white matter densities in brain areas associated with number processing. Heidekum et al. employed surface-based morphometry in a large sample of adults. They found associations of cortical surface properties such as sulcal depth with numerical intelligence, complex arithmetic ability, and higher-order mathematical knowledge.

Two studies investigated more applied aspects of (numerical) learning using tablet-based applications. Kohn et al. evaluated their adaptive training program Calcularis 2.0, which can be used to support dyscalculic children. The training yielded improved arithmetic and spatial number processing skills after only 12 weeks, which were still observable 3 months post-training. Jung, Meinhardt et al. evaluated a tablet-based assessment of early visuospatial abilities (VSA) using the application (app) MaGrid®. Their results substantiated the hypothesized factor structure of VSA proposed in the taxonomy of Newcombe and Shipley (2015) and provide evidence for a hierarchical development of VSA as assessed using MaGrid®.

A comparatively larger set of contributions investigated the foundations of mental arithmetic. The first three studies addressed the distinction between number magnitude manipulation (e.g., involved in calculation or number magnitude comparison) and arithmetic fact retrieval (e.g., as in simple multiplication). In a brain stimulation study employing rTMS, Fresnoza et al. investigated the role of the horizontal intraparietal sulcus (assumed to be involved in magnitude processing) and the left angular gyrus (assumed to be involved in arithmetic fact retrieval). The authors found that the involvement of these brain areas seemed to be modulated by the solution strategy employed (i.e., retrieval vs. calculation) rather than by the arithmetic operation *per se* (i.e., subtraction vs. multiplication). This means that solving less well-established multiplication problems was rather associated with the magnitude network, while solving highly overlearned subtraction problems was associated with the retrieval network. This interpretation fits well with the behavioral results of Jung, Moeller et al. Using a hemifield paradigm, the authors investigated a possible left lateralization of the representation of arithmetic facts in a number bisection task. The authors did not observe such a lateralization. They hypothesized that this might be due to the complexity of the task, so that participants might not have relied solely on arithmetic fact retrieval. However, the third study in this collection, by Suárez-Pellicioni et al. seems to contradict the notion that simple subtraction problems are solved by retrieval. The authors found in a longitudinal study using functional magnetic resonance imaging (fMRI) that children do not switch to retrieval when subtractions become more and more overlearned. Instead, it seemed that procedures became more automatic with skill development.

Another subset of studies investigated the neuro-cognitive correlates of arithmetic learning. Mosbacher et al. employed frontal and parietal anodal transcranial direct current stimulation (tDCS). While stimulation did not lead to an overall improvement of arithmetic performance, the authors observed that only frontal stimulation accelerated training gains. Additionally, Belkacem et al. compared abacus experts and non-experts with regard to their arithmetic performance and EEG activity. While the groups shared some neural signatures, they found differences indicating that abacus experts developed a new computational pathway by assigning number representations onto an imaginative abacus representation, which involved parallel activation of calculation-related areas.

Finally, in a theoretical contribution, Testolin elaborated on how computer simulation at cross-disciplinary intersections can help understand how numerical concepts are learned by the human brain. Although deep learning models are not yet designed to simulate higher-level mathematical thinking, they have the potential to grasp the acquisition of numerical concepts in its full complexity.

The broad range of studies presented in this collection documents the significant progress made in understanding different aspects of numerical cognition and development. The various methods used are state of the art and testify to the expertise of the researchers involved. The current Research Topic brought together expertise of researchers from different backgrounds which clearly advanced our understanding of numerical cognition and development and has the potential to contribute to processes of learning and teaching numerical and mathematical skills.

## Author Contributions

EK and KM drafted the manuscript. All authors read, corrected and approved the final manuscript.

## Conflict of Interest

The authors declare that the research was conducted in the absence of any commercial or financial relationships that could be construed as a potential conflict of interest.
